# Development of a novel nanoindentation technique by utilizing a dual-probe AFM system

**DOI:** 10.3762/bjnano.6.205

**Published:** 2015-10-12

**Authors:** Eyup Cinar, Ferat Sahin, Dalia Yablon

**Affiliations:** 1 Microsystems Engineering, Rochester Institute of Technology, USA; 2 Electrical and Microelectronic Engineering, Rochester Institute of Technology, USA; 3 SurfaceChar LLC., Sharon, MA, USA

**Keywords:** atomic force microscopy (AFM), mechanical characterization, nanoindentation

## Abstract

A novel instrumentation approach to nanoindentation is described that exhibits improved resolution and depth sensing. The approach is based on a multi-probe scanning probe microscopy (SPM) tool that utilizes tuning-fork based probes for both indentation and depth sensing. Unlike nanoindentation experiments performed with conventional AFM systems using beam-bounce technology, this technique incorporates a second probe system with an ultra-high resolution for depth sensing. The additional second probe measures only the vertical movement of the straight indenter attached to a tuning-fork probe with a high spring constant and it can also be used for AFM scanning to obtain an accurate profiling. Nanoindentation results are demonstrated on silicon, fused silica, and Corning Eagle Glass. The results show that this new approach is viable in terms of accurately characterizing mechanical properties of materials through nanoindentation with high accuracy, and it opens doors to many other exciting applications in the field of nanomechanical characterization.

## Introduction

Nanoindentation is a commonly used technique to estimate mechanical properties of materials. An indenter probe fabricated with a known tip geometry is used to penetrate into the sample. By utilizing the force and small amount of depth information measured during indentation, material properties such as elastic (Young’s) modulus of the sample can be estimated. For example, a growing application of nanoindentation is to determine the mechanical properties of cells which may be of critical importance for progressive diseases such as cancer or vascular diseases [1]. A recently published work by Guz et al. also investigates nanoindentation experiments on cell mechanics and proposes new models for determining the elastic modulus of cells [2]. In addition to the biomedical engineering field, nanoindentation has been widely used in many other disciplines where accurate mechanical characterization is of high importance [3–4].

The improvement of sensor technology has enabled the integration of higher resolution depth and force measurement techniques for nanoindentation tools. Although this helped in increasing the accuracy of the experimental data, the current research demonstrates that there are still limitations on the commercially available tools and various problems still need to be tackled [5–8].

Nanoindentation experiments requiring very low force values and high resolution usually use a standard AFM system. With this setup, an AFM cantilever probe is used for indenting the material and the probe displacement is monitored by laser beam bounce technology also known as optical lever method. With this methodology, a laser beam is reflected off the back end of the cantilever and directed towards a quadrant photodiode detector that monitors both vertical and lateral motion [9]. Force–distance (FD) curves can be generated based on displacement data and the spring constant value of the cantilever. Depending on the type of the material, various models can be applied in order to interpret and extract the elastic modulus of materials.

One of the problems with this AFM-based approach is cantilever bending. Most of the conventional AFM nanoindentation probes have spring constants below 500 N/m. Depending on the material hardness, the applied load could result in bending of the cantilever. With optical lever method, the displacement is measured by laser deflection, which includes laser deflection caused by both the indentation depth (motion in *Z*) and the cantilever bending (motion in *X* and *Y*). The convolution of *X* and *Y* motion into the measurement cause overestimation errors in the interpretation of material properties using FD curves.

Instrumented nanoindentation (INI) tools can be used for a large dynamic force range. However, the displacement and force sensitivity are significantly low as compared to AFM-based nanoindentation. Cohen et al. compare the two techniques and present the drawbacks of INI tools in terms of load and displacement sensitivity [10]. While typical INI tools have a load sensitivity on the order of 10 nN, AFM-based nanoindentation can have sensitivities of 0.05 nN or better [10]. Similarly, displacement sensitivity of INI tools is significantly lower than the AFM-based tools. Especially when an INI tool is used, due to the hardware limitations on both displacement and load sensing, the indenter probe might already have penetrated into the sample by the time surface contact is detected. This also yields critical errors in estimating the mechanical properties of materials as it was addressed in the literature by [6,8]. A final limitation in current AFM-based nanoindentation experiments is the use of lasers to monitor displacement data since there are various environments such as high vacuum or low temperature environments where the laser operation is complicated.

Considering the above mentioned problems, significant research has been devoted to the design and the development of tools that will improve the accuracy of the obtained experimental data and yield a more accurate estimation of material properties by nanoindentation. Evan et al. report the development of a tool specifically designed for nanoindentation on compliant materials considering the surface detection problems of commercially available nanoindentation devices [11]. Nowakowski et al. demonstrate a nanoindentation system with high precision where capacitive gauges are used for displacement measurement in the system [12]. The proximity of the indenter to the surface is sensed by tuning forks through their frequency response shift, showing the capabilities of accurate point of contact detection measurement of tuning forks. Oiko et al. recently demonstrated the development of nanoindentation probes that can be manipulated inside a scanning electron microscope (SEM) [13]. This system also utilizes tuning-fork technology, which can be used as an ultra-sensitive force sensor owing to the very high quality factors of tuning forks. They perform in situ nanoindentation experiments on multi-walled carbon nanotube bundles, however, the displacement data is only obtained from the SEM images limiting the high accuracy of displacement reading and the true depth sensing during nanoindentation. Zhao et al. present a nanoindentation device that is designed to operate inside an SEM chamber in order to perform in situ indentation tests of indium phosphide [14].

We report a novel approach using a multi-probe scanning probe microscopy (SPM) system with tuning-fork probe technology in an effort to overcome the limitations and problems of current high resolution nanoindentation systems such as AFM-based systems. Different than cantilever displacement measured by optical means, our approach uses a secondary AFM probe that is kept in closed-loop feedback contact with the indenter probe. This gives ultra-sensitive and high resolution capability in terms of true depth sensing during nanoindentation. With this approach, only the *Z* axis motion of the straight indenter is monitored, independent of any possible tuning fork bending that may occur in spite of the very large spring constant of tuning forks (above 4000 N/m). During nanoindentation of the specimen, the point of contact can be determined with great accuracy as compared to other nanoindentation tools since the positioning of the tuning forks is controlled with phase feedback. This is also an advantage for the experimentally obtained data and overcomes the major problem as discussed in [6,8].

In our previous work [15], we have presented the initial results of our approach by employing dynamic force determination techniques only. In this study, we extend our work on multi-probe and apply it to estimation of elastic properties for different types of materials by measuring the bending of the indenter tuning fork with an another AFM probe.

The rest of the paper is organized as follows. Firstly, a theoretical background of nanoindentation is introduced to the reader. Then, an overview of the proposed system is demonstrated with a detailed component descriptions of the whole multi-probe system. In addition, the overall system calibration data and procedures are introduced. In the fourth section, the results of nanoindentation experiments obtained on multiple calibration materials are introduced and compared with other references in the literature. The last section is devoted to the conclusions.

## Theoretical background on nanoindentation

**Figure 1:**
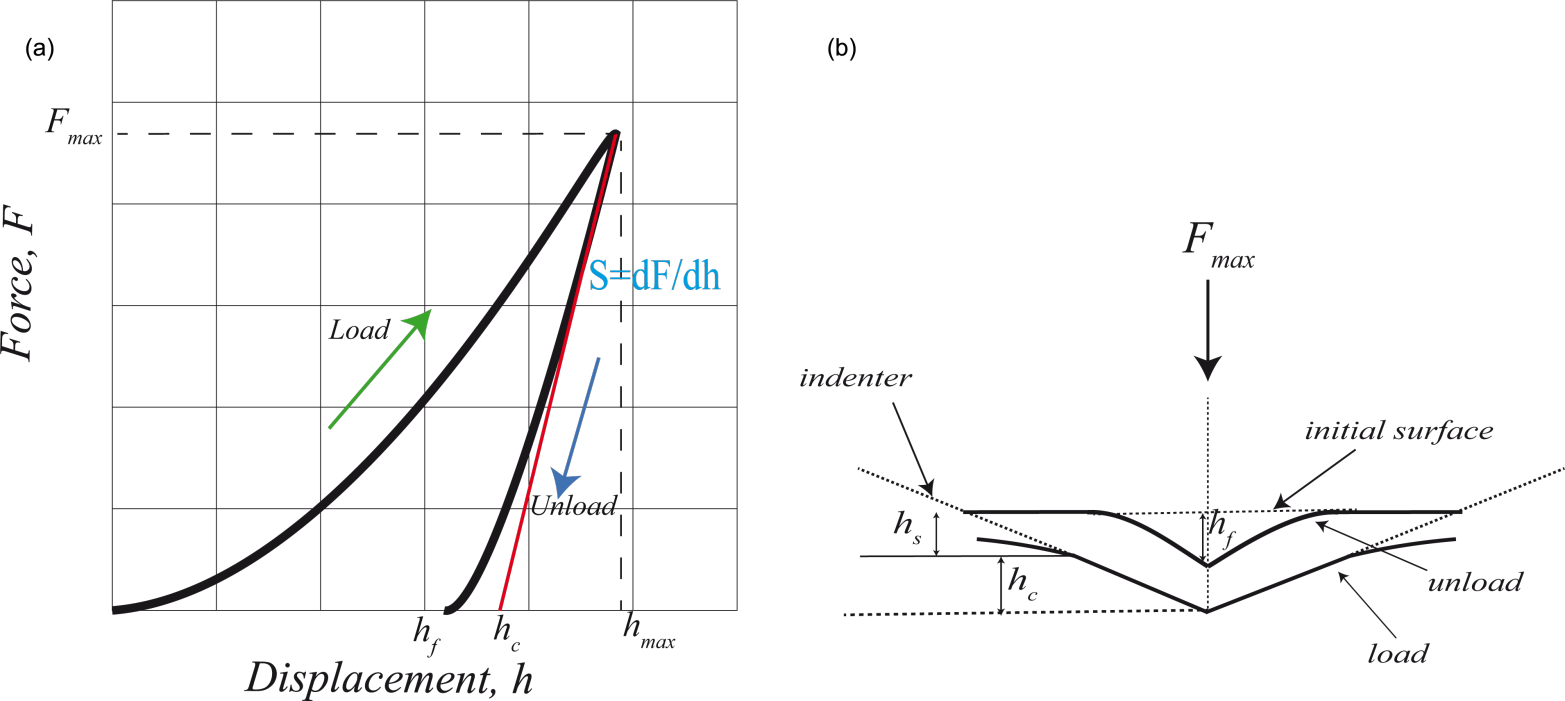
Schematic representation of the OP model. (a) A representative force–distance curve for the OP model; (b) Schematic illustrating loading and unloading process.

A widely used mechanical model in nanoindentation experiments is the Oliver–Pharr (OP) model [16]. Properties such as elastic modulus or hardness of materials can be extracted from force–distance curves using the OP model. Figure 1a shows a typical force–distance curve when nanoindentation includes a plastic deformation. In this curve, the loading part includes both elastic and plastic deformation. However, during the unloading portion, it is assumed that only elastic deformation occurs. Therefore, stiffness can be approximated with the slope of unloading curve as shown in Figure 1a. If the unloading curve is fit to a power law such as *F* = α(*h* − *h_f_*)*^m^* where α and *m* are power-law fitting constants then the unloading stiffness *S* can be approximated as in Equation 1 by the slope of the fitting.

[1]
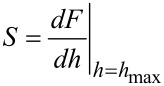


Figure 1b shows the important parameters during the nanoindentation process. The depth at contact between indenter and substrate is *h_c_*, *h_s_* is the sink-in of the material during indentation and *h_f_* is the final depth of penetration that is left on the surface after nanoindentation is completed. Once a force curve such as that in Figure 1a is obtained, one can calculate elastic unloading stiffness through Equation 2 defined as the slope of the upper part on the unloading curve as shown in Figure 1a:

[2]
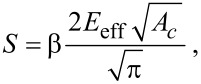


where *E*_eff_ is effective elastic modulus including both the elastic modulus of the indenter (*E*_1_) and of the sample elastic modulus (*E*_2_). It can be expressed as given in Equation 3. β is a correction factor that accounts for lack of axial symmetries for the indenter. It has been shown by Oliver and Pharr that β ≈ 1.07 worked for most of the materials.

[3]
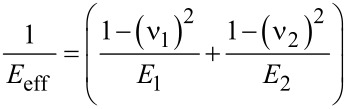


The projected contact area of elastic contact, *A_c_* in Equation 2 depends on both indenter’s tip geometry and the depth of contact, *h_c_*. It is possible to establish a mathematical form for the area function such as *A_c_*(*h_c_*) based on the specific tip geometry of the indenter.

Once a force–distance curve is obtained such as shown schematically in Figure 1a, one can calculate the stiffness parameter *S* from the slope of the unloading part and use Equation 2 and Equation 3 to extract the unknown elastic modulus of the sample (*E*_2_).

In the next section we introduce an overview of the proposed system and its components in detail. We also present the calibration data and the parameters that have been used for the rest of the experimental results.

## Overview of the novel multi-probe nanoindentation system

**Figure 2:**
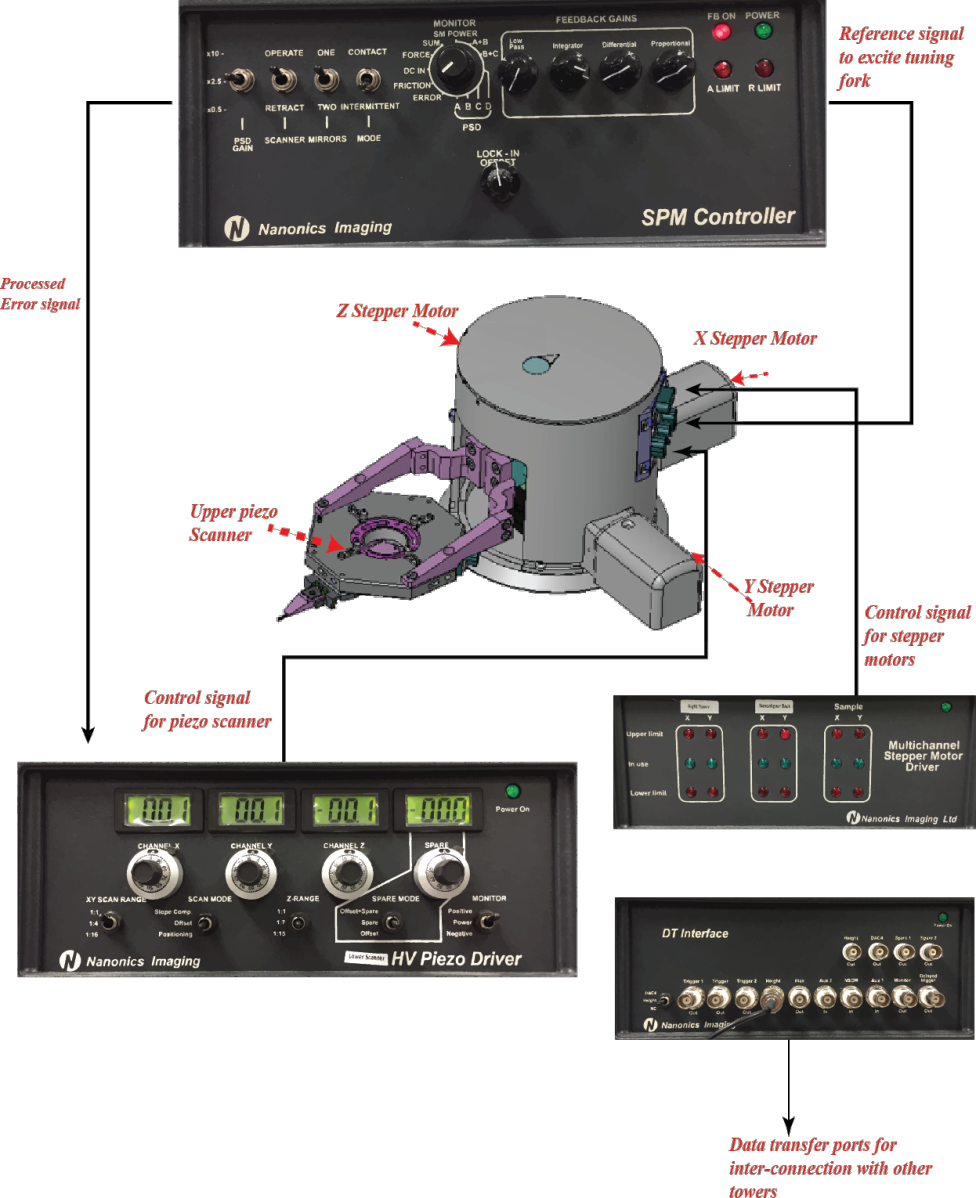
SPM control units and interconnections for a single tower system [17].

The proposed system uses a multi-probe SPM instrument (Nanonics MultiView-4000) based on normal force tuning fork technology. The tuning forks have a resonance frequency of approximately 34 kHz and a high Q factor in air that is over 1000. The instrument consists of four towers where each tower has lateral stepper motors for *XYZ* motion as shown in Figure 2 with a resolution of 21 nm. Each tower has also an upper piezo scanner integrated together with a pre-amplifier block, which amplifies the signal received from the tuning fork. The upper piezo scanners are used for probe scanning and have a range of 80 μm (*XYZ*) and a resolution <0.05 nm in *Z* direction and <0.15 nm in the *XY* directions. In addition to the upper piezo scanners, there is also an independent lower piezo scanner that holds the sample holder and has a range of 80 μm in all directions.

The tuning fork is tuned to its resonance frequency, and either the oscillation amplitude or phase can be used for feedback. Based on the amplitude or phase feedback error, each tower can be independently controlled in a closed-loop feedback. The SPM controller box, shown in Figure 2, controls the feedback mechanism during a scan based on its lock-in amplifiers and PID controllers. Tuning forks are oscillated with 5 V amplitude signal and piezo scanners are controlled with high voltage signals (±145 V) generated by the high voltage (HV) piezo driver box shown in Figure 2. The oscillation frequency is based on the resonance frequency of each tuning fork. Based on the error signal obtained either through phase or amplitude feedback, the tip is moved so that a constant error signal is established that keeps the probe tip in continuous contact with the sample.

Data exchange between two towers can be established by using data transfer (DT) interface boxes shown in Figure 2 and BNC cables. These data transfer interface boxes provide the user interface for signal exchange (input/output) between data transfer digital acquisition (DAQ) cards of each tower. For example, the height information that is read from one tower can be transferred to the other tower through auxiliary input ports of the destination tower data transfer interface box. Each tower is connected to a separate PC that runs the proprietary control and scanning software for the multi-probe AFM system. A detailed system analysis of the multi-probe SPM system is given in [17].

**Figure 3:**
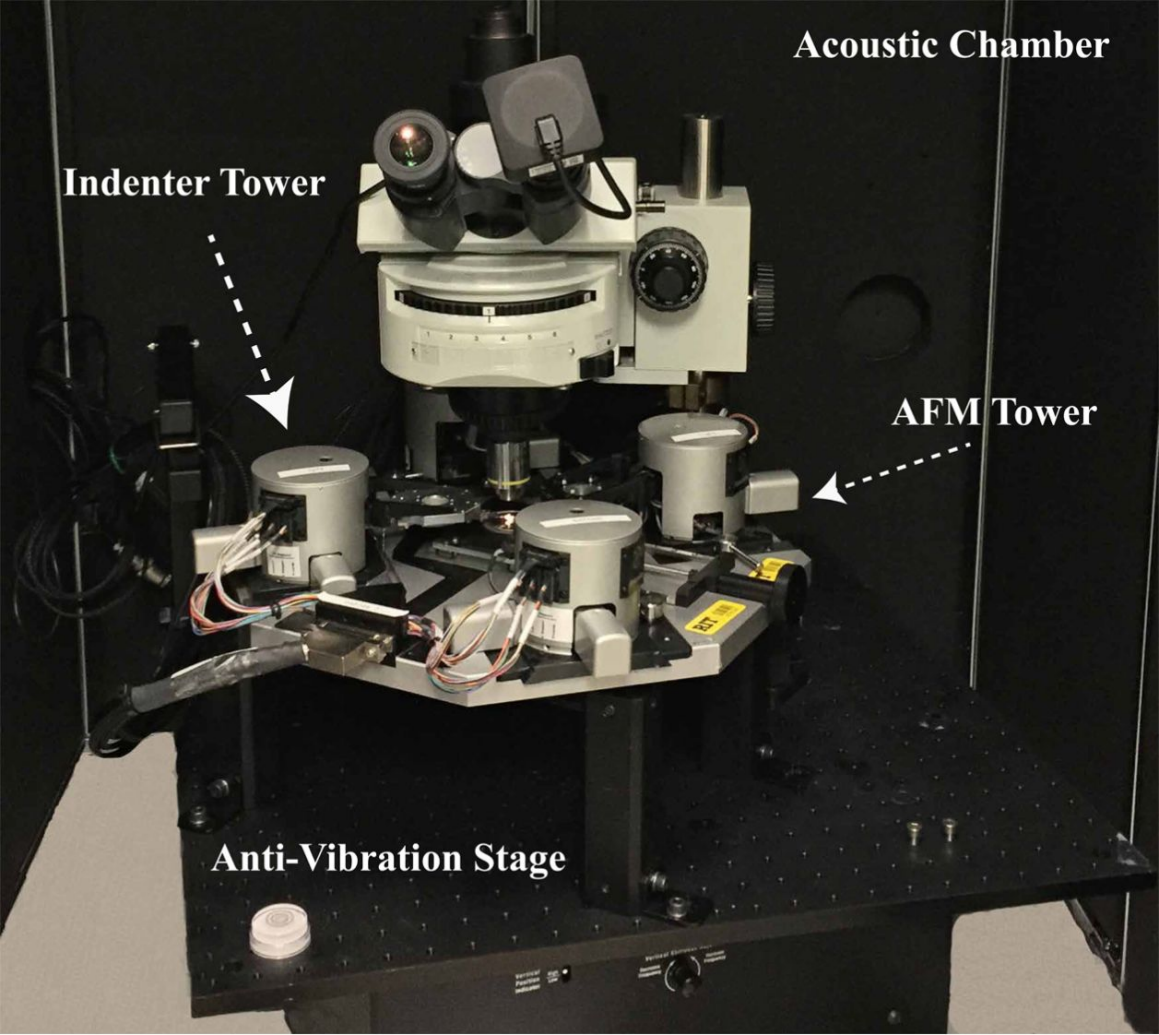
Positioning of the multi-probe system inside the acoustic chamber.

Figure 3 shows the positioning of multi-probe system. There are four towers and a sample piezo scanner stage which is positioned in the middle. Each tower can be operated independently. The towers and the scanners sit on an anti-vibration table and the entire setup is enclosed within an acoustic chamber to eliminate acoustic noise.

**Figure 4:**
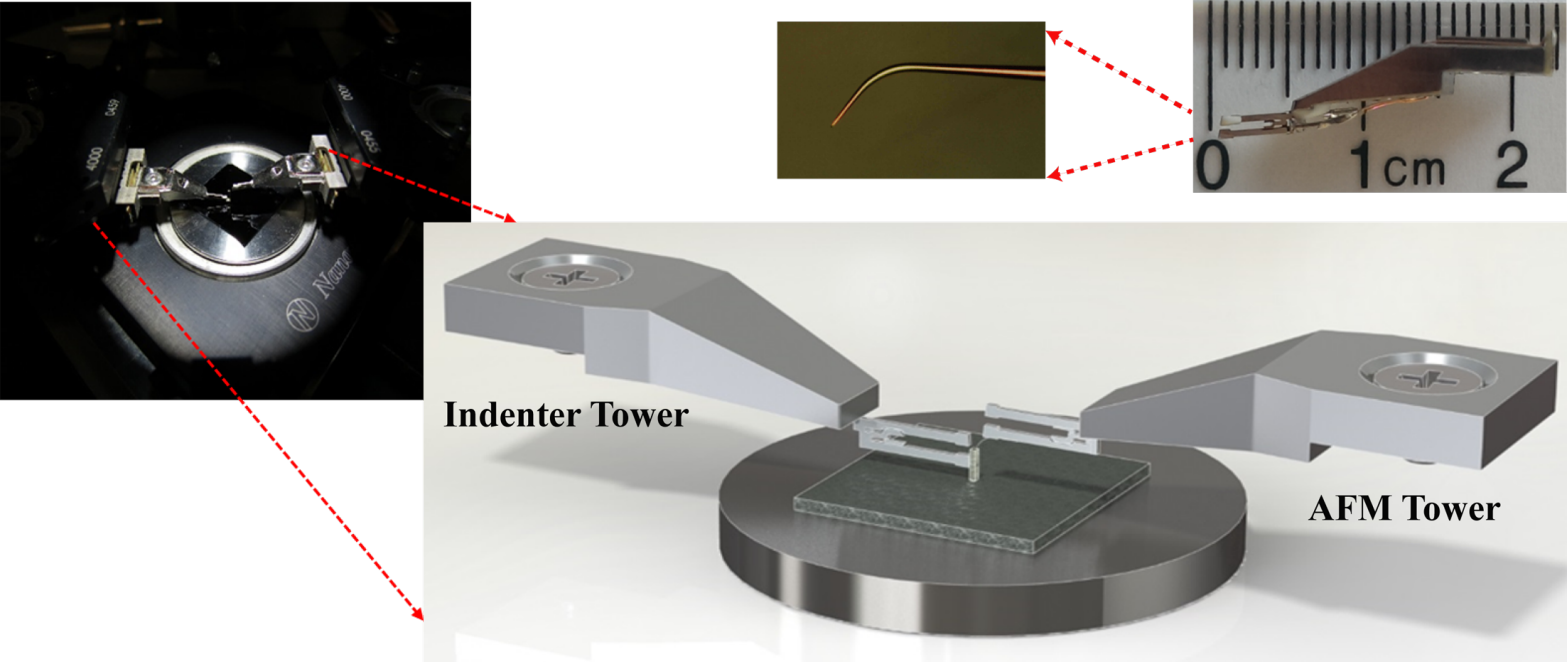
Experimental setup demonstrating the proposed two probe nanoindentation technique.

Figure 4 includes the details of the experimental setup demonstrating the multi-probe nanoindentation technique. For nanoindentation experiments, a diamond probe with a cube-corner geometry (MicroStar Technologies) is mounted by Nanonics Imaging Ltd. (Jerusalem, Israel) so that there is no tilt of the indenter probe tip relative to the surface. During the mounting process of the indenter tip, a special mounting tool is used to mount the tip normal to the surface and accordingly guarantee the angle of the tip relative to the fork and the angle of the fork relative to the mount. Penetration depth is measured by the second tower with a specifically fabricated cantilevered AFM glass probe tips coated with Cr. These probes have a cantilever length of 300 μm and 20 nm tip radius, and they are mounted onto the lower tine of the tuning forks.

An example image of cantilevered AFM tips is also shown in Figure 4. The material under investigation is placed on a flat sample holder on the lower piezo scanner. Initially, the left hand side probe (indenter tower probe) is moved down towards the sample by the *Z* stepper motors while phase feedback error signal is monitored continuously. This means that the feedback is based on the change of the phase signal from the tuning fork. Contact of the tip with the sample is observed immediately with a change in error signal, at which point the stepper motion stops. Subsequently, the lower scanner is retracted by a safe distance amount (≈3 μm). After this, with the help of the fine movement capability of the piezo scanner, the sample is brought up automatically to ensure a very accurate contact positioning and a safe approach. Once the indenter probe is in feedback with the sample surface, the probe is held in that state and the second probe on the right hand side (AFM tower probe) is placed on top of the diamond probe. The positioning of the two probes is shown schematically in Figure 4. Once the contact is established for both probes, the nanoindentation experiment is started.

For nanoindentation experiments, the indenter probe oscillation is disabled and the desired sample displacement value is set. For example, for a target of 100 nm displacement, the programmed sample scanner first retracts the sample 100 nm and then pushes toward the indenter probe for 200 nm. The displacement of the indenter is monitored with the AFM probe, which is oscillating and kept in phase-feedback. In our proposed system, the depth sensing is performed with an AFM probe which is in phase-feedback with the top of the diamond indenter probe. The height of the AFM probe is controlled with a piezo scanner head which has a very high resolution (*<*0.05 nm) due to highly oriented piezo materials used in the folded-piezo flexure scanner design. This also brings the ultimate resolution to our nanoindentation experiments in terms of depth sensing. In addition, since the AFM probe continuously monitors the Z-axis displacement of the indenter probe, only changes in Z motion are sensed with a very high accuracy of point of contact while the X and Y motion are ignored.

**Figure 5:**
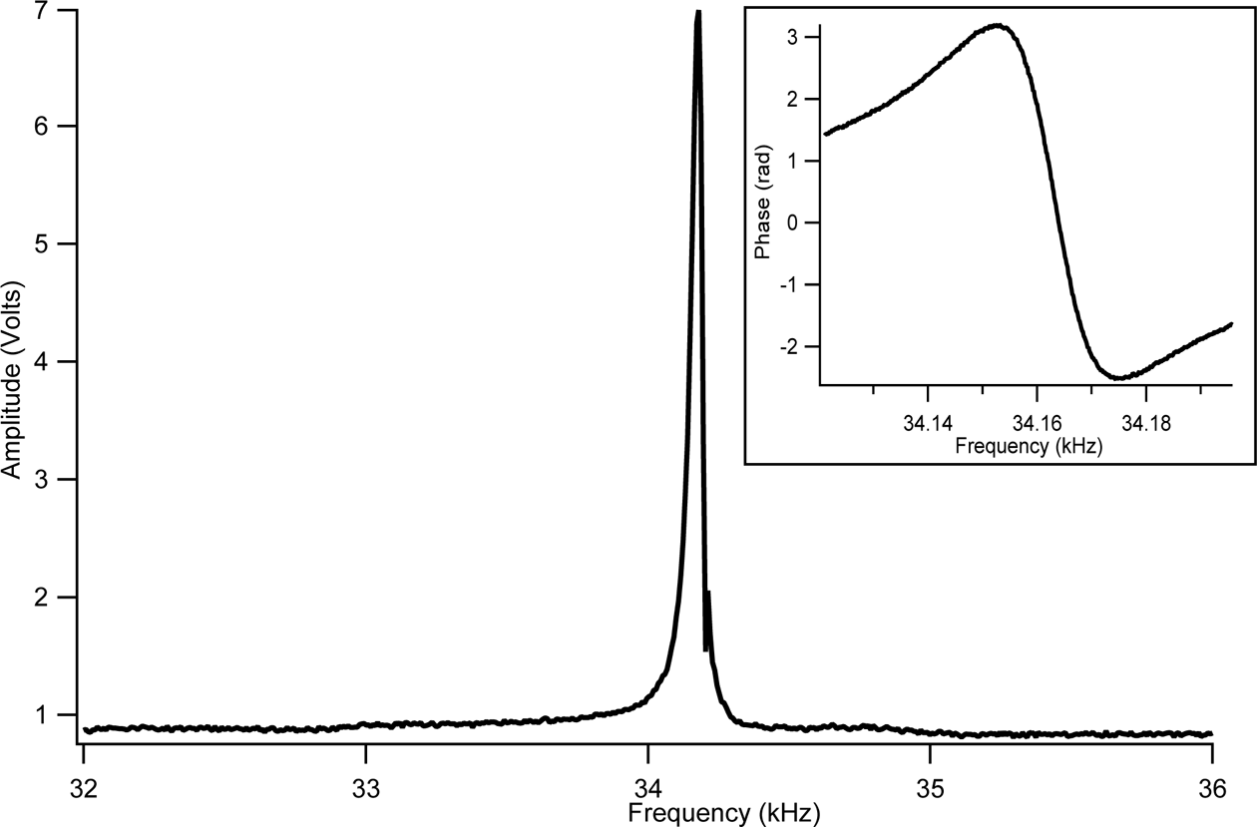
An example resonance response of AFM probes used in the experiments.

Figure 5 shows a representative AFM probe frequency–amplitude response curve with an inset including the change of phase over frequency. As the plots indicate, the tuning fork probes have a very sharp resonance curve enabling a sensitive error signal with accurate closed-loop feedback control. Depending on the application, it is possible to configure the system to be able to work in either amplitude or phase feedback error based on the oscillation of the tuning fork. Considering the rapid change of phase as shown in Figure 5, a phase feedback is more sensitive compared to the amplitude feedback. With the help of a built-in lock-in amplifier system, it is possible to monitor both the amplitude and the phase of oscillations. When the phase feedback is used, the amplitude of oscillation can be independently monitored.

Calibration of the height signal is performed with a calibration grid (BudgetSensors) that includes both 115 nm micropillar and microwell arrays on a silicon chip. The step height of the features on the chip is measured and verified by using both contact profilometer and SEM measurements.

**Figure 6:**
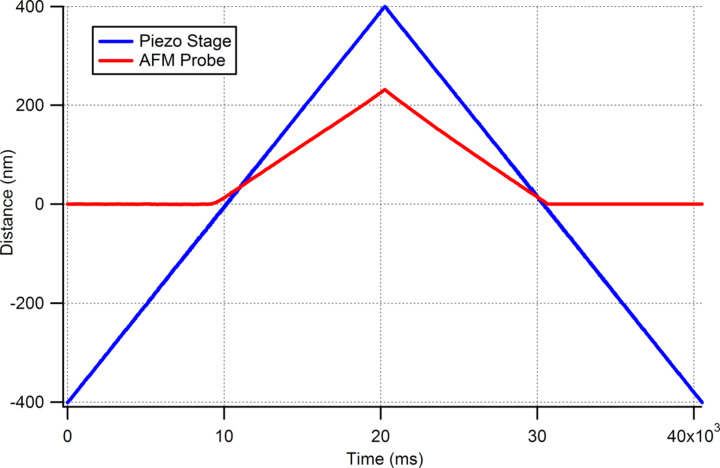
Experimental data showing AFM probe measurements on top of the diamond indenter for a fused silica sample.

Figure 6 shows the data collected during a multi-probe indentation experiment on a fused silica sample. Initially, the diamond probe approaches the surface in phase feedback. When the contact is detected based on phase error signal, the approach is stopped automatically. The PID gains are then re-adjusted so that the error signal stays at zero with minimum deviation when the probe is in contact. After this, the approach of the AFM probe is initiated to the top of the indenter. Similarly to the indenter probe, the AFM probe is operated in phase-feedback mode. When contact is established with the indenter probe, the approach is stopped automatically and PID settings are adjusted so that the AFM probe will remain in contact at all times and will just follow the movement of the indenter. After the contact for both probes is established, the configuration of the stage movement in the software is performed. In Figure 6, the stage is programmed to move ±400 nm. When the indentation process is started, the stage retracts 400 nm first and then moves towards to the indenter. During the motion of the sample stage towards the indenter, the indenter probe oscillations are turned off and the indenter probe is no longer in feedback. The second set of data in red shown in Figure 6 is the displacement data read from the AFM probe during the indentation process. Note that the AFM probe reflects only the true *Z* axis movement of the indenter probe unlike the conventional AFM systems where the measurement relies on laser deflection of the cantilever itself which includes a convolution of *X* and *Y* motion into the laser deflection reading.

As it can be seen from Figure 6, as the piezo stage starts pushing up the sample towards the diamond indenter, the reading of the AFM probe starts to go up as well and when the sample starts moving away from the diamond tip, the AFM probe reading starts going down and settles at position zero when the sample and the indenter are totaly dissociated.

For the force level calculations, the spring constant of the tuning fork can be calculated from the beam formula as given in Equation 4:

[4]
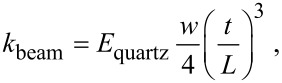


where *w* and *t* are the width and the thickness of the free prong, respectively and *L* is the length of the prong. *E*_quartz_ is elastic modulus of the quartz material of which the tuning forks are fabricated. Several studies have found this formula to be inaccurate and underestimate the spring constant of the tuning forks by a significant amount, even up to an order of magnitude [18–19]. One reason for this is that the tip (diamond indenter in our case) is rigidly fixed at the end by epoxy glue and this might alter the effective dimensions of the free beam. Therefore, further investigation of the spring constant calculation based on this model is necessary.

**Figure 7:**
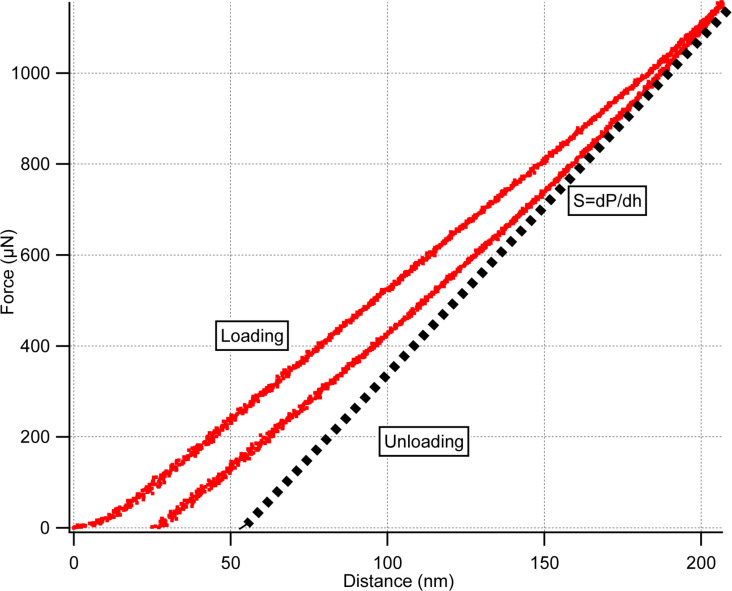
Fused silica force–distance curve.

We derive the effective spring constant of the indenter tuning fork by calibrating against a sample with known modulus. Based on the formula given in Equation 4 and the dimensions of a bare tuning fork, the spring constant is calculated as 2600 N/m. Firstly, an indentation experiment is performed on fused silica by using *k*_fork_ = 2600 N/m. Then, Oliver–Pharr (OP) model is utilized to match the experimental data to the known elastic modulus of fused-silica sample 69.3 GPa [16,20]. Fitting of the data over 10 different force-curve measurements with an average modulus of 69.38 GPa yields a spring constant *k*_fork_ = 4992 ± 264.11 N/m. One of the force–distance curves used in OP model fitting is given in Figure 7.

Table 1 tabulates the results of the calibration experiment on the fused silica sample. Where *h*_max_ represents the maximum depth of penetration and *K*_calibration_ is the spring constant value of the indenter tuning fork.

**Table 1:** Experimental data obtained during calibration of *k*_fork_ on the fused silica calibration sample.

*h*_max_ (nm)	*K*_calibration_ (N/m)	estimated modulus (GPa)

113.8	4850	69.6
157.6	5041	69.32
159	5041	69.47
159.4	5034	69.32
159.9	4490	69.38
160.6	5180	69.31
161.3	5175	69.29
165.5	5450	69.35
170.3	4620	69.4
206.98	5035	69.35

mean (std)	4992 (±264.11)	69.38 (±0.09)


**Figure 8:**
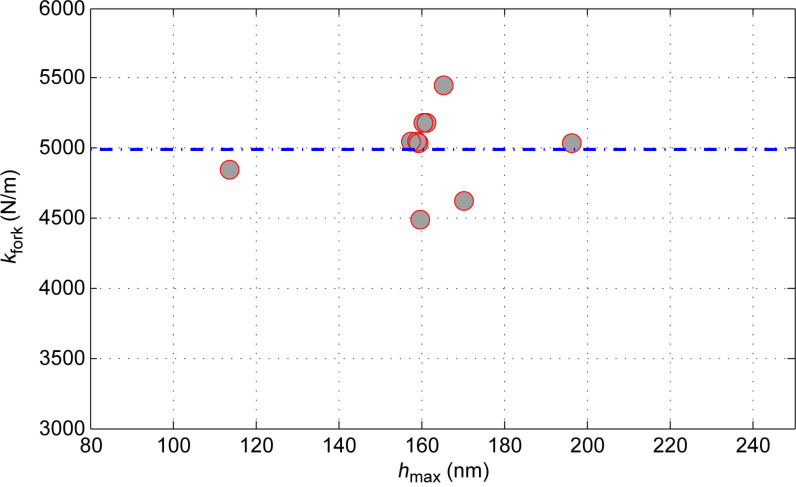
Spring constant vs maximum depth of penetration.

Figure 8 demonstrates the distribution of spring constant values with respect to depth of penetration during fused silica calibration experiments. The mean value of the spring constant is shown with a dotted line at 4992 N/m.

**Figure 9:**
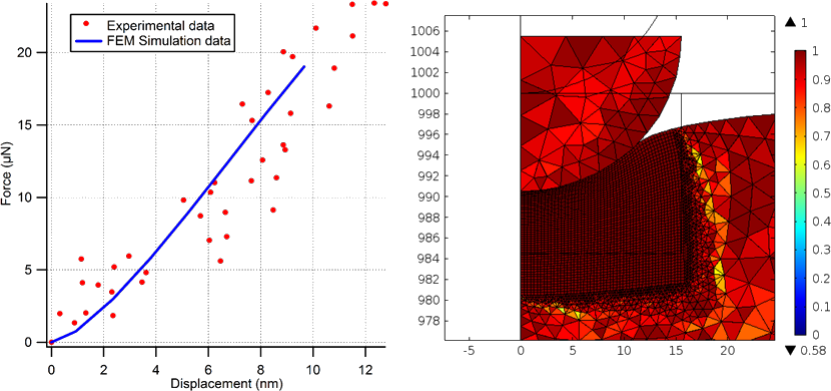
Finite element analysis data as compared to experimental data.

To further verify the spring constant calibration and the force values for the rest of the experiments, finite element (FE) simulations have been performed as shown in Figure 9. In the simulations, the diamond indenter is pressed into the fused silica reference sample incrementally up to 10 nm depth of penetration in order to ensure that the results stay within the elastic regime. During the simulation, reaction forces on the surface of the indenter tip are evaluated showing that force levels for both experimental and simulation data are in excellent agreement when *k*_fork_ is 4992 N/m.

**Figure 10:**
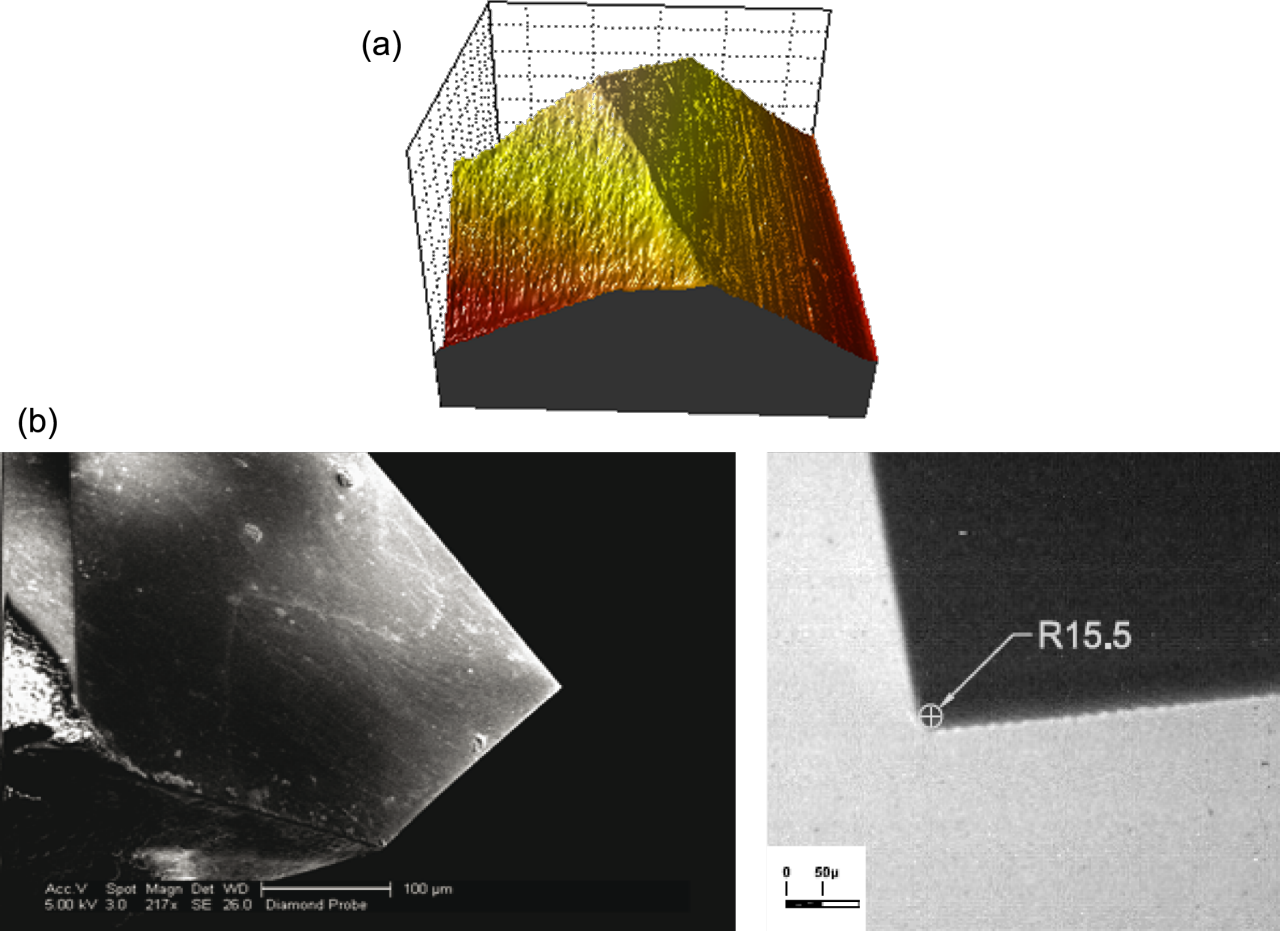
Images of cube-corner diamond tip used in nanoindentation experiments. (a) A 3D representation of AFM scan for the cube-corner diamond tip used in nanoindentation experiments. (b) SEM and TEM images of the diamond tip. The tip radius is measured as 15.5 nm.

Figure 10a shows the characteristics of the cube-corner geometry diamond indenter tip used in our nanoindentation experiments. Figure 10a is obtained from an AFM scanning with the AFM tower and shows the cube-corner shape of the tip. Figure 10b is an TEM image of the tip showing the radius of curvature in nanometers.

In the next section, we present the results obtained with other types of materials and our analysis of elastic modulus estimations showing the viability and the reproducibility of the proposed technique.

## Results and Discussion

In the previous section, we have introduced the overall system components and the details of our proposed technique together with the calibration results. In this section, we present the nanoindentation results on different materials and our estimations based on the experimentally obtained data.

**Figure 11:**
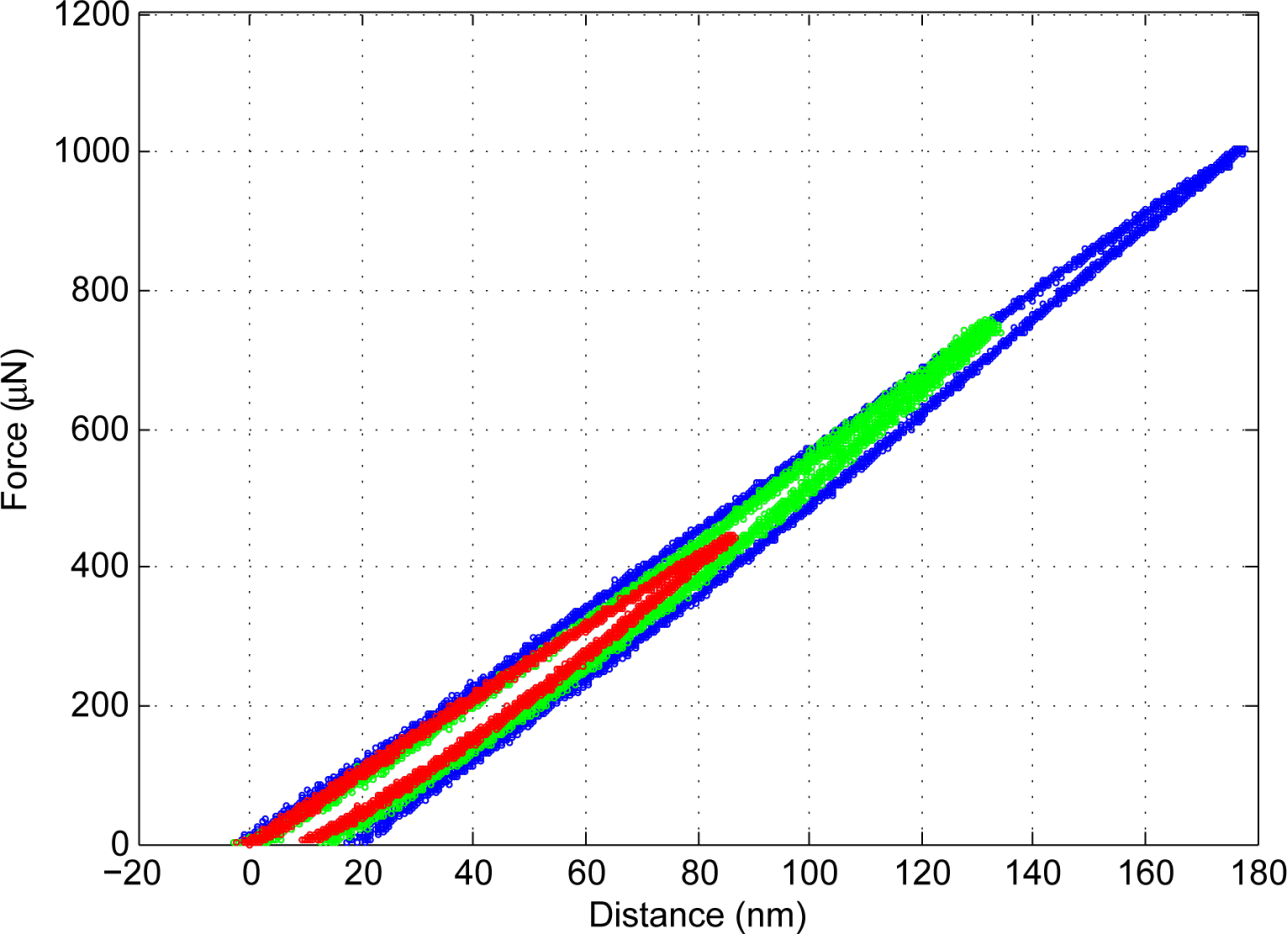
Force–distance curves on silicon substrate.

Figure 11 includes the force–distance curves with varying loads measured on silicon (100). The varying loads correspond to the programmed stage movements from 100 to 300 nm. Within these experiments, the stage moves with a speed of 0.04 nm/ms. The maximum force increases from 445 to 1004 μN.

**Figure 12:**
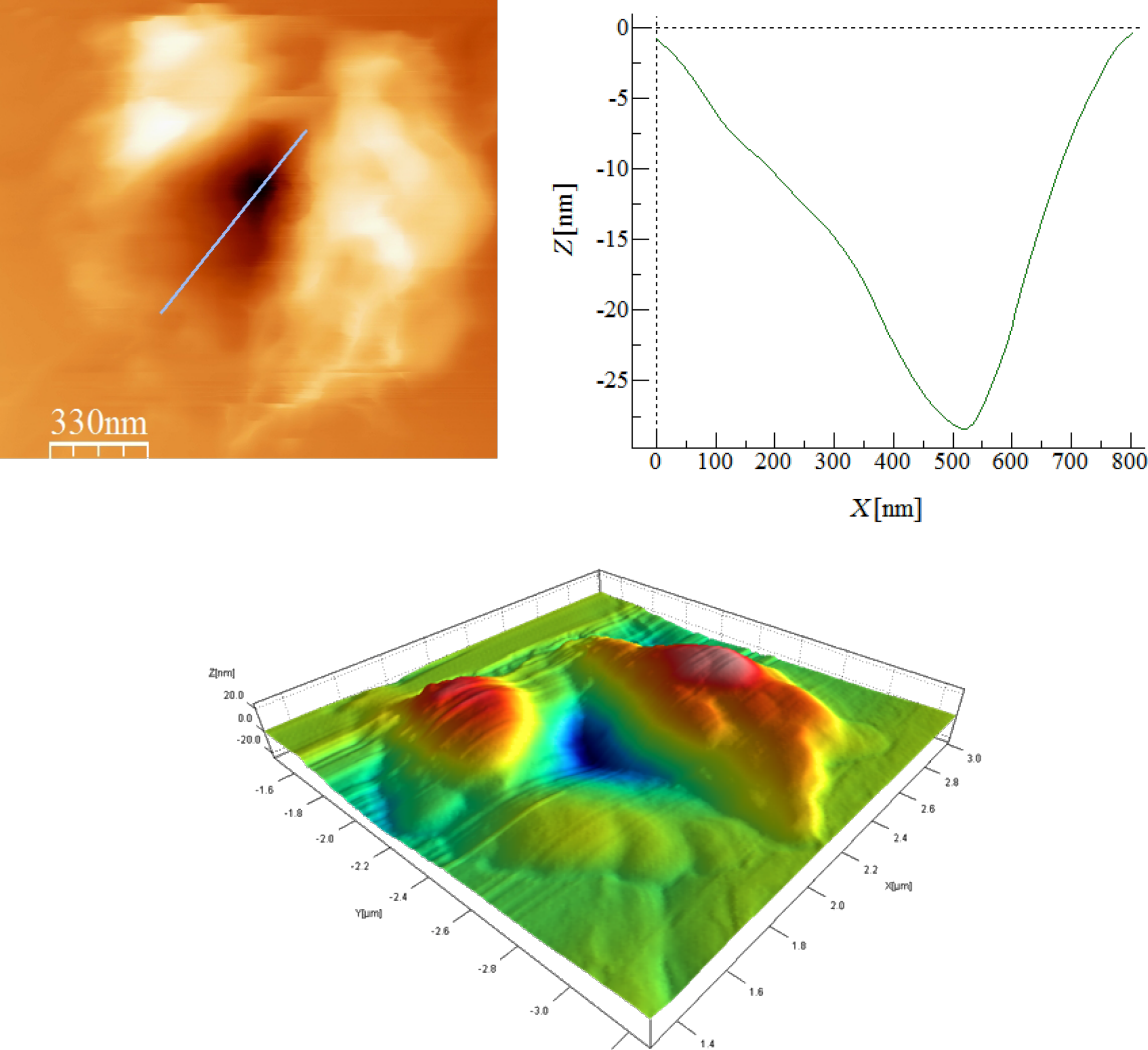
Topography and cross-sectional profile of indent on silicon.

An AFM topographical image of indentation on silicon substrate is shown in Figure 12 together with its height profile. It shows quantitatively a residual indentation mark of the cube-corner indenter tip at a depth of 27.5 nm. The overlapping pattern of force–distance curves together with the residual cube-corner tip indentation mark normal to the surface show the effectiveness of our technique with negligible probe twisting artifacts.

Table 2 shows the properties of the materials used in nanoindentation experiments in our study and their corresponding material properties reported in the literature.

**Table 2:** Reported properties of the materials used in nanoindentation experiments.

material	*E* (GPa)	Poisson ratio	reference

Si(100)	169	0.22	[21]
fused silica	69.3	0.17	[20]
Eagle Glass™	70.9	0.23	[22]
diamond (tip)	1150	0.07	[16]

**Figure 13:**
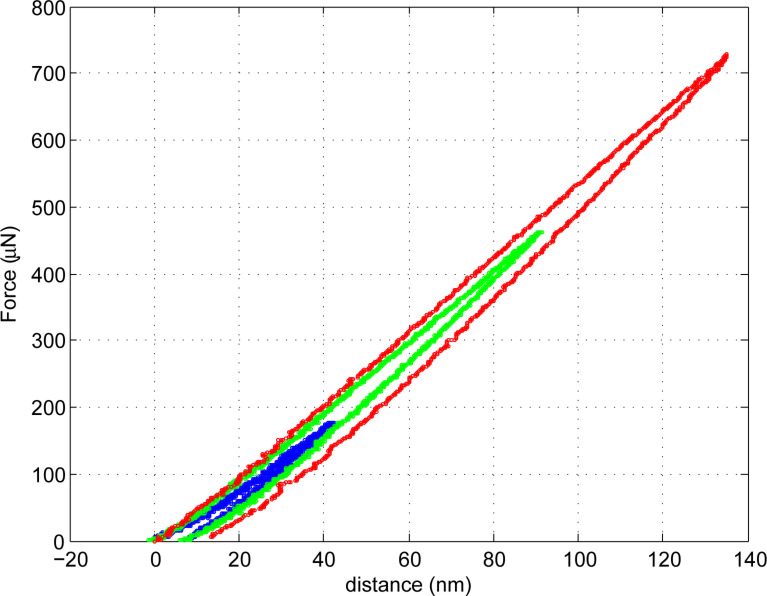
Force–distance curves on Corning Eagle Glass substrate.

Figure 13 shows the nanoindentation results on a glass substrate (Corning^®^ Eagle Glass 2000™) with an elastic modulus of 70.9 GPa and varying load conditions similar to silicon nanoindentation experiments.

**Figure 14:**
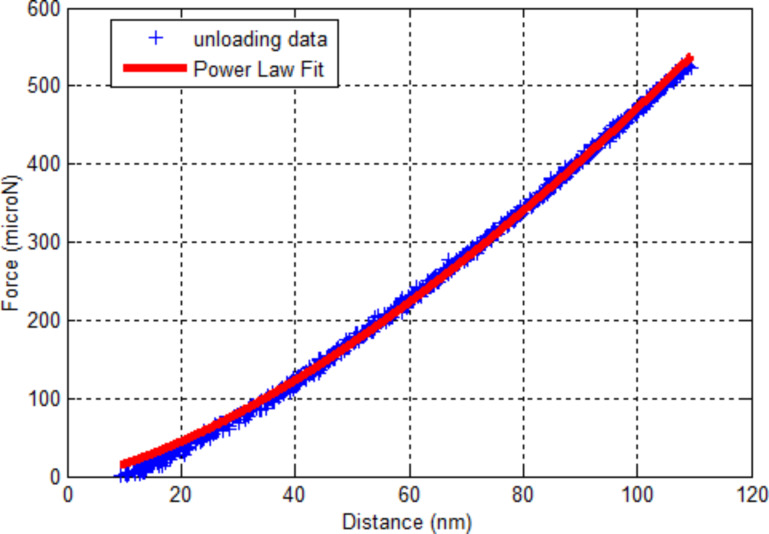
Power-law fitting to the unloading part of a silicon force–distance curve.

Figure 14 shows an example of a power-law fitting of the unloading data from a experimentally obtained silicon force–distance curve. The fitting parameters α = 0.6668 and *m* = 1.4668 fall within the expected ranges as listed in the OP model [16].

In our calculations, the area function is taken as *A*(*h_c_*) = 2.598001*h_c_*^2^. This is based on considerations about physical measurements on the tip and the geometric calculations of the cube-corner indenter as suggested by the manufacturer’s data sheet of the diamond-tip.

The results in Table 3 present the experimentally obtained elastic modulus estimations of the materials used in this study. Mean values are taken over ten different set of experiments for each sample at varying load levels. Compared to reported moduli values in the literature, the values shown in Table 3 are in a good agreement.

**Table 3:** Estimation of elastic modulus by Oliver–Pharr fitting and the parameters used.

materials	estimated modulus (GPa)	reported modulus (GPa)	Oliver–Pharr β parameter

Si (100)	166.87 ± 27.42	169	1.04
fused Silica	69.38 ± 0.09	69.3	1.04
Eagle Glass	67.83 ± 7.68	70.9	1.04

## Conclusion

We present the development of a novel approach to nanoindentation by using a multi-probe SPM system. The new approach brings ultra-high resolution to nanoindentation experiments in terms of both the force and depth sensing. The second AFM probe monitors only the true *Z* axis motion as the straight indenter probe is lifted in the *Z* direction. This is a significant improvement over conventional AFM-based nanoindentation experiments that convolute *XY* motion into *Z* motion with laser-based detection of cantilever motion. Additionally, the use of a tuning fork gives excellent force sensitivity due to its significantly higher spring constant, quality factor, and ability to track motion through phase feedback. Our experimental results show that this system measures the material properties, accurately.

In addition to an indenter probe and an AFM probe, with the current system up to four probes can be operated and could work in tandem. This opportunity brings other exciting novel applications to our nanoindentation approach. For example, while the two probes are performing a nanoindentation experiment, the third and the forth probe can be used in identifying changes in other material properties on-line. By attaching a conductive Pt nanowire probe tips, the third probe can be used as a voltage source and the fourth probe can be used to measure the current. In this way, electrical nano-characterization of the sample can be performed on-line during nanoindentation. Furthermore, thermoresistive probes can be integrated into the approach to monitor the thermal properties of the material on-line during nanoindentation. Lastly, this novel approach can be integrated into environments where the usage of lasers is not possible such as the case in conventional AFM nanoindentation experiments.

## Acknowledgements

The authors would like thank to Nanonics Imaging Ltd. for collaboration in the development of this novel application.
